# Tumor and bone marrow uptakes on [^18^F]fluorodeoxyglucose positron emission tomography/computed tomography predict prognosis in patients with diffuse large B-cell lymphoma receiving rituximab-containing chemotherapy

**DOI:** 10.1097/MD.0000000000008655

**Published:** 2017-11-10

**Authors:** Chin-Chuan Chang, Shih-Feng Cho, Hung-Pin Tu, Chia-Yang Lin, Ya-Wen Chuang, Shu-Min Chang, Wen-Ling Hsu, Ying-Fong Huang

**Affiliations:** a Department of Nuclear Medicine, Kaohsiung Medical University Hospital; b Institute of Clinical Medicine, Kaohsiung Medical University; c Division of Hematology & Oncology, Department of Internal Medicine, Kaohsiung Medical University Hospital; d Department of Public Health and Environmental Medicine, College of Medicine; e Department of Medical Imaging and Radiological Sciences, Kaohsiung Medical University, Kaohsiung, Taiwan.

**Keywords:** bone marrow, diffuse large B-cell lymphoma, FDG PET/CT, prognosis, survival

## Abstract

The purpose of this study was to determine the relevance of standardized uptake value (SUV) on [^18^F]fluorodeoxyglucose (FDG) positron emission tomography/computed tomography (PET/CT), focusing on tumor and bone marrow, to disease outcomes based on progression-free survival (PFS) and overall survival (OS) in patients with diffuse large B-cell lymphoma (DLBCL) receiving rituximab-containing chemotherapy.

We reviewed the records of patients with DLBCL who were diagnosed between September 2009 and January 2013 and underwent pretreatment whole-body FDG PET/CT scans. All patients received rituximab-containing chemotherapy. The maximal SUV of tumor (SUVt) and maximal SUV of sternum (SUVst) were measured. Univariate and multivariate analyses were used to assess the prognostic significance of SUVt, SUVst, gender, age, clinical stage, international prognostic index (IPI), and laboratory tests.

There were total 70 patients enrolled in this study. The median follow-up time was 36 months. An SUVt cut-off value of ≥19 had the best discriminative yield for PFS (*P* = .04). An SUVst cut-off value of ≥1.6 had the best discriminative yield for OS. The 3-year OS rates for patients with maximal SUVst < 1.6 and for those with maximal SUVst ≥1.6 were 74.8% and 57.1%, respectively (*P* = .04). Further forward, multivariate Cox proportional hazards model revealed that maximal SUVst (hazard ratio: 2.62; 95% confidence interval: 1.10–6.28; *P* = .03) and IPI were significant factors affecting OS.

In patients with DLBCL receiving rituximab-containing chemotherapy, elevated maximal SUVt ≥19 was an independent predictor for shorter PFS, and maximal SUVst ≥1.6 was an independent predictor for shorter OS. It adds the value of pretreatment FDG PET/CT scans.

## Abstract 2

Supplemental Digital Content is available in the text

## 1 Introduction

Diffuse large B cell lymphoma (DLBCL), accounting for up to 30% of newly diagnosed cases in the western countries,^[1]^ is the most common subtype of non-Hodgkin lymphoma (NHL) and is characterized by its diversity in morphology, immunophenotype, cytogenecity, and clinical course. Because it is an aggressive form of NHL and patients exhibit a wide range of outcomes due to the heterogeneous entity, complete pretreatment evaluation including prognostic classification is important for disease management.

In the past 20 years, the international prognostic index (IPI) has been one of the most useful tools to evaluate patient prognosis in the clinic.^[2]^ In recent years, however, the addition of rituximab, a chimeric IgG1 monoclonal antibody targeting CD20, to chemotherapy has led to a marked improvement in clinical outcomes.^[3]^ For most patients with newly diagnosed DLBCL, a combination of rituximab and chemotherapy is now the standard treatment. For patients who are at a high risk of relapse, however, more intensive chemotherapy, upfront autologous stem cell transplantation (ASCT) after the first remission, or other newly developed drugs may be required to improve survival instead of combination chemotherapy with rituximab.^[4,5]^ Defining prognostic factors with new prediction tools has been thus a cornerstone to classify patients into an appropriate risk group before treatment and can help differentiate patients who could benefit from more aggressive chemotherapeutic regimens.

Positron emission tomography/computed tomography (PET/CT) using [^18^F]fluorodeoxyglucose (FDG), the most popular radiotracer used for PET scan, is gaining popularity and plays an important role for the diagnosis, staging, and therapeutic monitoring in patients with DLBCL.^[6–8]^ As a glucose analog, the uptake of FDG in the tissue closely correlates with the glucose utilization and reflects tissue metabolism. Interest in using FDG PET/CT to evaluate prognosis has increased because uptake of FDG in tumors is proportional to the metabolic rate of viable tumor cells and may reflect the aggressiveness of the tumor.^[9]^ Recently, metabolic tumor volume (MTV) on FDG PET/CT has been widely used as a prognostic parameter to predict patient prognosis, and has been shown to be more predictive than the Ann Arbor staging system^[10,11]^ and for patients with bone marrow involvement.^[12]^ The MTV on interim FDG PET also showed predictive value for patients with DLBCL.^[13]^ However, the relationship between bone marrow uptake on pretreatment FDG PET/CT and disease outcome has been poorly studied.

Previous studies regarding nonsmall cell lung cancer^[14]^ and squamous cell carcinoma of the head and neck^[15]^ have been shown that patient with higher bone marrow uptake on FDG PET has poorer survival. In our clinical experience, we have noticed that some patients, who underwent FDG PET/CT for pretreatment staging of DLBCL, showed increased bone marrow metabolic activity. We have also observed that patients with high standardized uptake value (SUV) on bone marrow seemed to have poor clinical outcome. However, this has not been confirmed by a systemic analysis of clinical cases. Therefore, the purpose of this study was to determine the relevance of SUV on FDG PET/CT, focusing on tumor and bone marrow, to disease outcomes based on progression-free survival (PFS) and overall survival (OS) in patients with DLBCL.

## 2 Materials and methods

### 2.1 Patients

We retrospectively analyzed the medical records of patients with DLBCL who were diagnosed between September 2009 and January 2013 and treated at Kaohsiung Medical University Hospital, a tertiary medical center in Southern Taiwan. A whole-body FDG PET/CT scan for pretreatment staging was performed in all patients. Complete pre-treatment evaluation including history, physical examination, standard laboratory tests, as well as bone marrow aspiration and biopsy were collected. The exclusion criteria included under 18 years of age and patients with a known history of other malignancy. The study design and review process was approved by the Institutional Review Board [KMUHIRB-E(I)-20160009] of Kaohsiung Medical University Hospital. Patient consent was not required because all of the clinical data were retrospectively collected via medical chart review. However, informed consent upon admission for all of the medical procedures was required. Patients were staged according to the Ann Arbor staging criteria. The patients subsequently received combination chemotherapy with rituximab. The observation period was from September 2009 to December 2014.

### 2.2 Treatment and clinical course

All patients received rituximab-containing chemotherapy, that is, rituximab combined with cyclophosphamide, doxorubicin, vincristine, and prednisolone (R-CHOP), as an initial therapy. After completion of chemotherapy, involved field radiation therapy was administered for residual tumor or bulky disease (≥10 cm). Complete remission (CR) was defined by follow-up image evaluation either by FDG PET/CT or CT scan according to published criteria.^[16]^ Patients experiencing refractory and relapsed disease were treated with salvage chemotherapy or received ASCT with high-dose chemotherapy if clinically indicated.

### 2.3 FDG PET/CT acquisition

Before the examination, every patient had fasted for at least 6 hours. Their blood glucose level was controlled to be less than 150 mg/dL before the tracer injection. After intravenous injection of 370 MBq (10 mCi) FDG, patients were asked to lie comfortably to reduce unnecessary muscular uptake. Sixty minutes after administration of FDG, a whole-body FDG PET/CT imaging was obtained on the dual-modality PET/CT system (Discovery ST 16; the GE Medical System, Waukesha, WI). Initially, the spiral low-dose CT was performed with cranio-caudal direction for the purpose of precise anatomical localization and attenuation correction. Thereafter, the emission acquisition in a reverse direction was conducted. Nine to ten bed scans per patient were acquired depending on the patient's body height. Emission scan time per bed position was about 4 minutes. All images were taken in the supine position without intravenous contrast media administered. Then, the PET data were reconstructed iteratively (ordered-subset expectation maximization method) with attenuation correction after decay correction, and reoriented in the serial coronal, saggital, and transverse slices. A workstation (Xeleris Functional Imaging Workstation; the GE Medical System, Waukesha, WI) was used for image analysis, display, and visualization. The maximum-pixel intensity projection was also used for visual evaluation.

**Figure 1:**
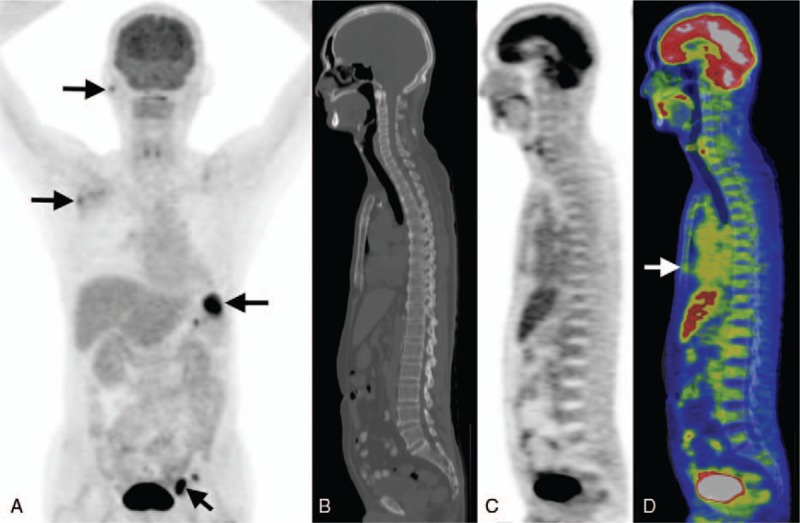
Demonstration of the FDG PET/CT image and the SUV measurement for FDG uptake of bone marrow. This 69-year-old man was diagnosed with stage III DLBCL. Maximal intensity projection (MIP) of the pretreatment FDG PET revealed multiple high grade FDG-avid lesions in the right parotid, right axillary, left inguinal regions, as well as the spleen (arrows in A). Using CT image for localization, the maximal SUVst was recorded by placing the ROIs over the sternum slice by slice (arrow in D). His maximal SUVt and SUVst were 15.1 and 1.78, respectively. Thereafter, he received treatment with R-CHOP regimen. However, the patient expired 10 months after diagnosis due to progression of the disease. A = MIP, B = CT, C = PET, D = fused PET and CT images.

Using CT images of the FDG PET/CT, the maximal SUV was measured by drawing a region of interest (ROI) over different foci. In patients with multiple lesions, the maximal SUV of tumor (SUVt) was obtained by placing the ROI over the most intense slice of the primary lesion. The FDG uptake of bone marrow is obtained by measuring the maximal SUV of sternum (SUVst), which was measured by placing the ROIs over the sternum slice by slice and then the maximal value was collected, as illustrated in Fig. 1.

### 2.4 Statistical analysis

PFS was defined as the time from diagnosis to relapse, progression, or death. OS was defined as the time from diagnosis to death from any cause. Continuous variables are presented as median (range). Continuous and categorical variables were analyzed using a Mann–Whitney *U* test or Chi-square test for comparisons between patients with high and low maximal SUVst, as appropriate. A Spearman rank correlation was used to determine the correlation between different variables. A Kruskal–Wallis test was used to compare the difference of maximal SUVst between patients at different clinical stages. The comparisons of survival curves were evaluated by Kaplan–Meier analysis. The univariate Cox proportional hazards model for survival were performed on maximal SUVt, maximal SUVst as well as on the accepted prognostic factors that were available from patient records [age, sex, staging, glutamate oxaloacetate transaminase (GOT), glutamate pyruvate transaminase (GPT), creatinine, albumin, lactate dehydrogenase (LDH), β2-microglobulin, and the hematological parameters such as hemoglobin, platelet and white blood cell (WBC) counts]. The cut-off values of the variables were dichotomized using normal reference reported in the literature when available, or selected by the receiver operating characteristic (ROC) curve, which yielded the most discriminative value. Variables shown to be of prognostic significance in the univariate analysis were then entered into a multivariate forward (Wald) Cox proportional hazards model. All these analyses were performed using SPSS statistical software, version 19.0 (SPSS, Chicago, IL). All statistical tests were 2-sided, and a 2-tailed *P* < .05 was considered significant.

## 3 Results

### 3.1 Patient characteristics

Seventy patients who met the eligible criteria were analyzed. There were 37 male and 33 female patients with age range from 20 to 94 years. Patients’ clinical characteristics including IPI factors, clinical, and laboratory parameters are listed in Table 1. Thirty-five (50%) patients were at an early stage (stage I and II), while the other 35 patients (50%) were at stages III and IV. According to IPI score, patients with low risk (0–1), low-intermediate risk (2), high-intermediate risk (3), and high risk (4–5) were 26, 26, 9, and 9, respectively. Thirty (42.9%) patients presented extranodal involvement at diagnosis. Seventeen (24.3%) patients had bone marrow involvement of lymphoma confirmed by bone marrow biopsy at the diagnosis. The median of maximal SUVt and maximal SUVst were 15.1 (3.0–38.2) and 1.49 (0.75–6.27), respectively. The median follow-up time was 36 months.

**Table 1:**
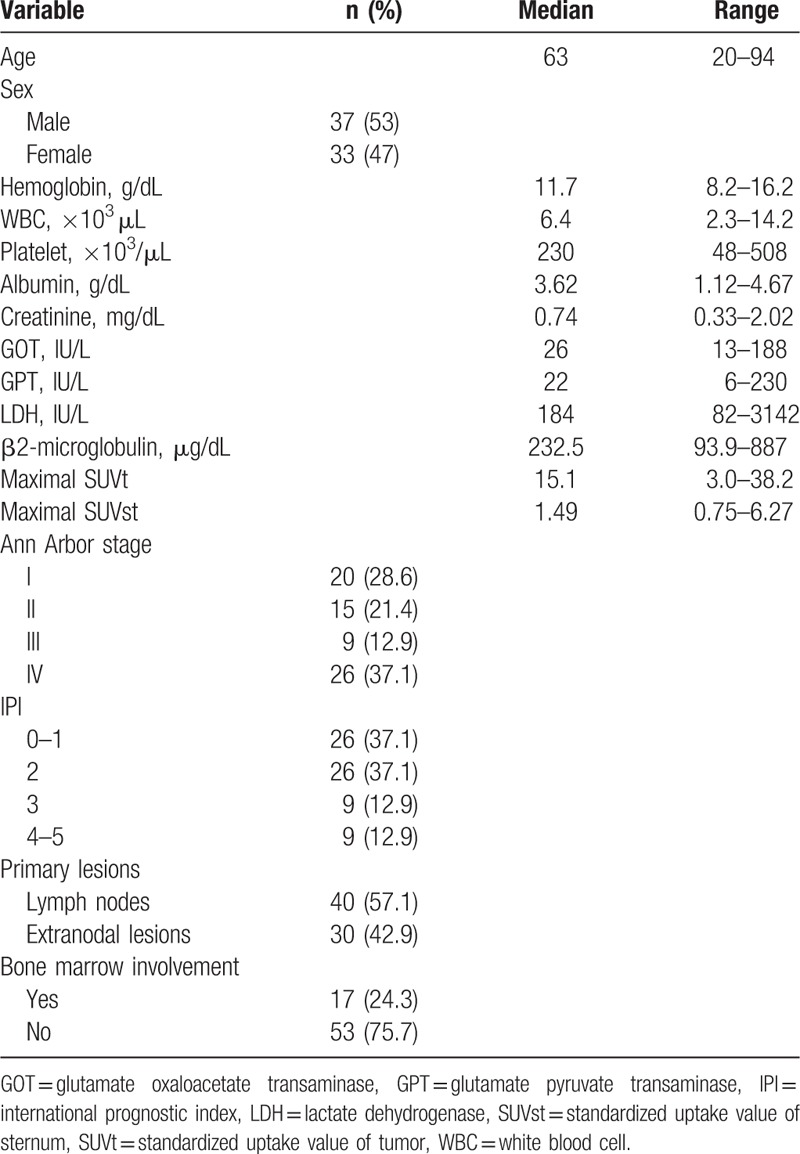
Characteristics at diagnosis of all 70 patients with diffuse large B-cell lymphoma.

### 3.2 Correlations between metabolic and clinical prognostic parameters

Using the Spearman rank correlation test, both maximal SUVt and maximal SUVst were not significantly related to clinical stage, bone marrow involvement by the tumor cell, IPI scores, hemoglobin level, white blood cell count, and platelet count (Table 2). On the Kruskal–Wallis test, there was no significant difference (*P* = .27) on maximal SUVst between patients at different clinical stages (see Figure, Supplemental Content).

**Table 2:**
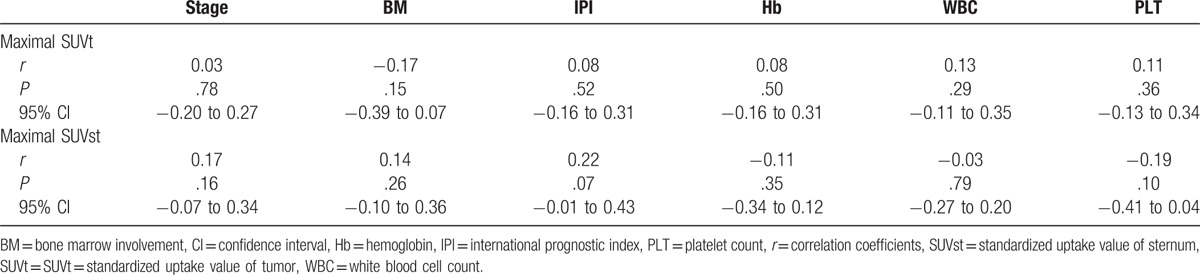
Correlations between maximal SUV and the clinical and hematological parameters.

### 3.3 Clinical outcomes according to different prognostic parameters

**Figure 2:**
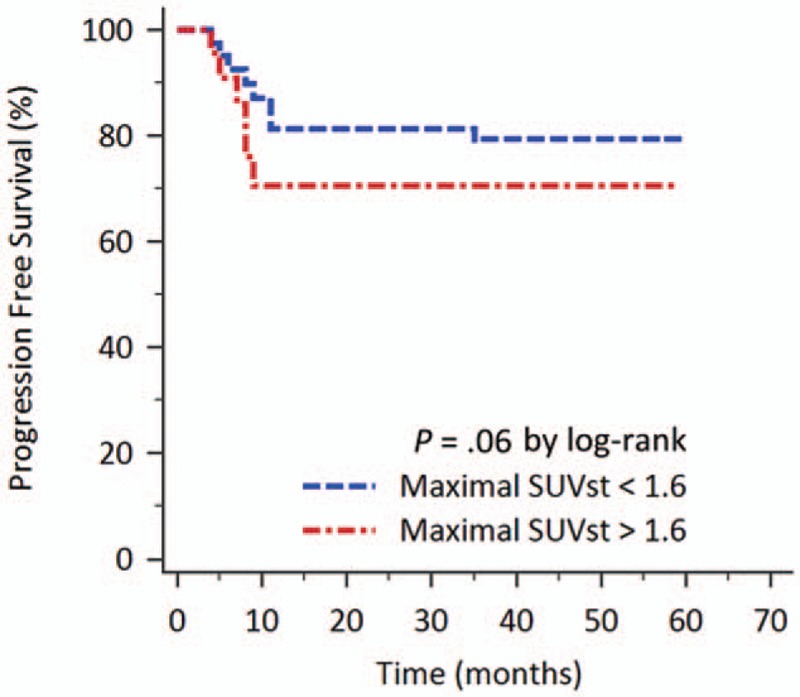
Kaplan–Meier curve of the patients according to the sternal FDG uptake value on PET/CT for PFS. Patients with maximal SUVst ≥1.6 had poorer PFS than patients with maximal SUVst < 1.6.

The correlation between patient survival and clinically collected parameters was compared. Using Kaplan–Meier survival analysis to evaluate PFS, patients with advanced clinical stage (*P* = .03), a higher maximal SUVt (≥ 19; *P* = .04), and a higher LDH level (≥192 IU/L, *P* = .03) had poorer PFS. Higher IPI scores and higher maximal SUVst correlated with poorer PFS, but analysis did not meet statistical significance (Fig. 2). Univariate analysis using Cox proportional hazard model yielded similar results. The following multivariate analysis of PFS showed that the high SUVt [hazards ratio (HR): 3.27; 95% confidence interval (CI): 1.11–9.60; *P* = .03] was independent poor prognostic factor (Table 3).

**Table 3:**
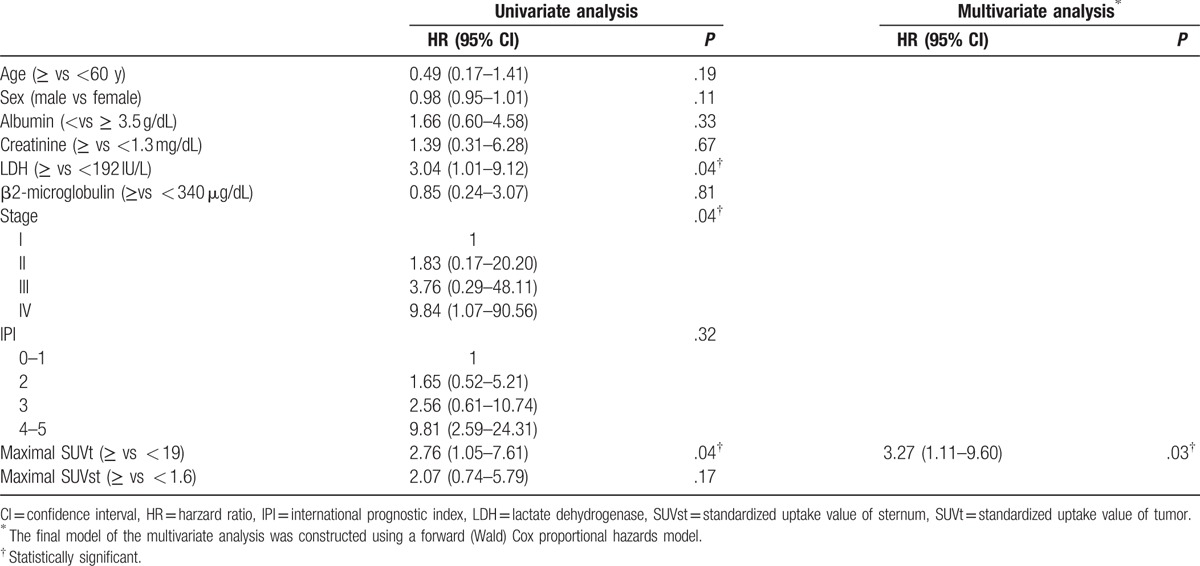
Cox proportional hazards model analysis of potential prognostic factors affecting progression-free survival.

**Figure 3:**
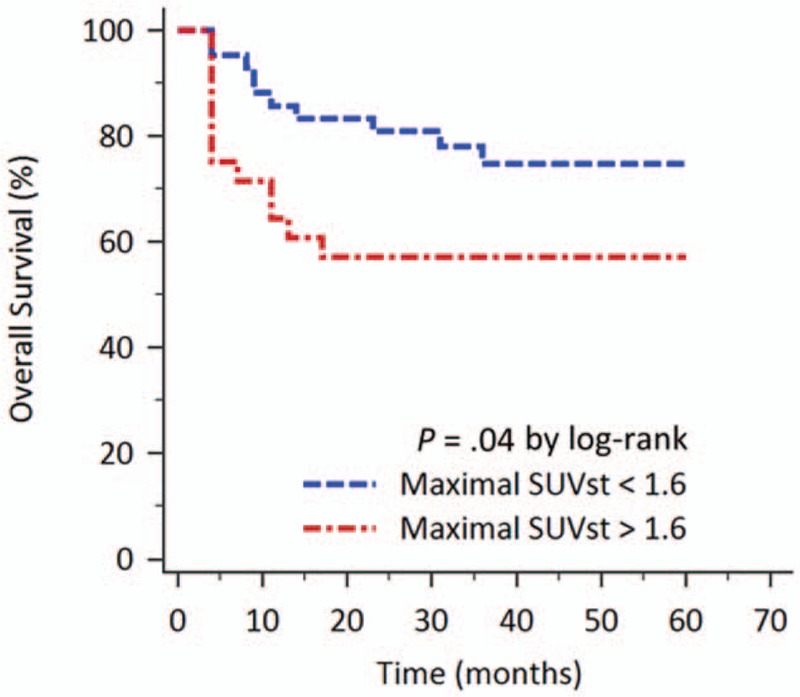
Kaplan–Meier curve of the patients according to the sternal FDG uptake value on PET/CT for OS. Patients with maximal SUVst ≥1.6 had poorer OS. The 3-year OS rates for patients with low maximal SUVst and for those with high maximal SUVst were 74.8% and 57.1%, respectively (*P* = .04).

As to the OS, the Kaplan–Meier survival analysis revealed that patient with high SUVst had poor OS than patients with low SUVst. The cut-off value of maximal SUVst for affecting OS was determined as 1.6 using the analysis of ROC curve (area under ROC curve 0.612, *P* = .14). The 3-year OS rates for patients with low maximal SUVst (<1.6; n = 42) and for those with high maximal SUVst (≥1.6; n = 28) were 74.8% and 57.1%, respectively (*P* = .04; Fig. 3).

### 3.4 Comparison between clinical characteristics in patients with low and high maximal SUVst

Clinical characteristics of patients with low maximal SUVst (<1.6) and those with high maximal SUVst (≥1.6) are summarized in Table 4. Both groups had similar backgrounds regarding age, gender, stage, IPI classification, tumor presenting site, bone marrow involvement, hemoglobin level, and WBC counts. The recurrence rate of all patients was 20%. The recurrence rates of patients with low SUVst and those with high SUVst were 19.0% and 21.4%, respectively (*P* = .63). However, patients with high maximal SUVst had a significantly higher mortality rate than that of those with low maximal SUVst values (*P* = .005).

**Table 4:**
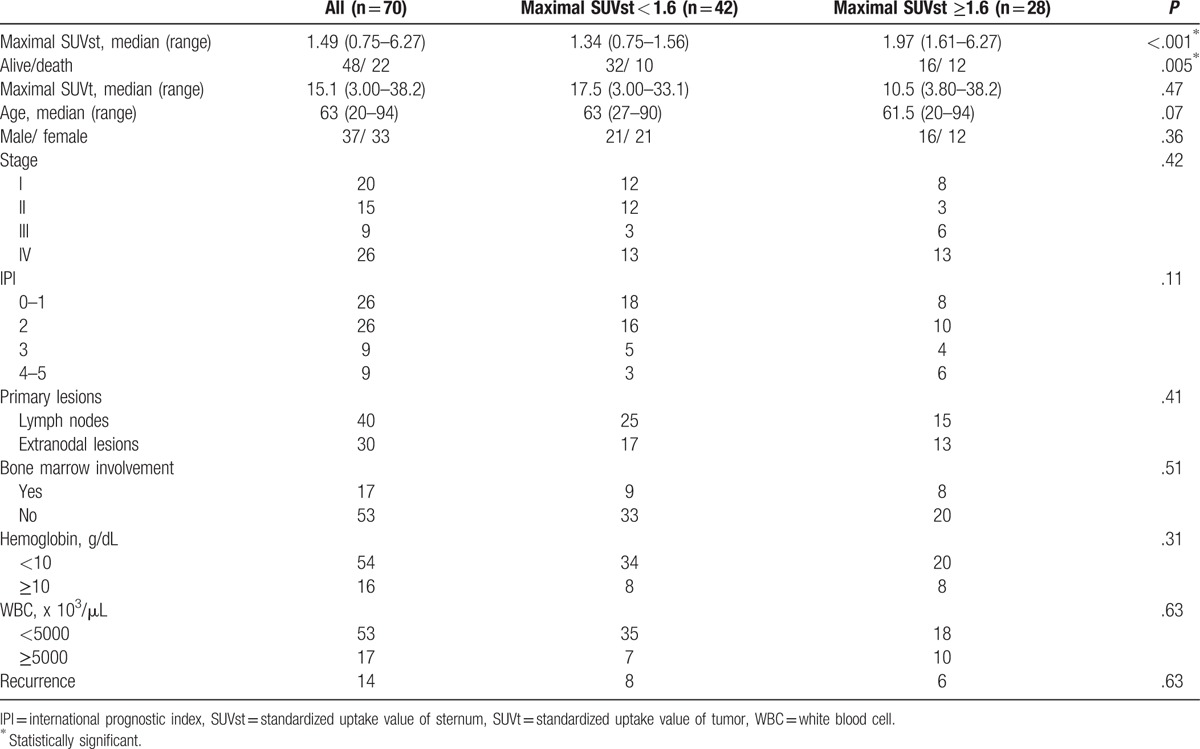
Comparison of clinical characteristics between patients with low and high sternal uptake on fluorodeoxyglucose positron emission tomography/computed tomography imaging.

### 3.5 Comparison of clinical impacts of other prognostic parameters

In univariate analysis (Table 5), patients with lower hemoglobin (<10 g/dL; *P* = .03), lower white blood cell count (<5000 x 10^3^/μL; *P* = .02), lower albumin level (<3.5 g/dL; *P* = .03), and higher LDH level (≥192 IU/L; *P* = .006) had poorer OS. Higher clinical stage (*P* = .02) and higher IPI risk score (*P* = .001) also correlated with poorer OS (Table 5). Then, the comparison of sternal FDG uptake and survival revealed that maximal SUVst ≥1.6 had significantly lower OS than patients with maximal SUVst < 1.6 (*P* = .04). Patients with thrombocytopenia, elevated GOT, GPT, β2-microglobulin, and high maximal SUVt also had poorer OS, but the results were not statistically significant. Further, multivariate analysis of OS using Cox proportional hazard models showed that the high IPI (HR: 2.3; 95% CI: 1.50–3.52; *P* = .002) and high maximal SUVst (HR: 2.62; 95% CI: 1.10–6.28; *P* = .03) were independent poor prognostic factors (Table 5).

**Table 5:**
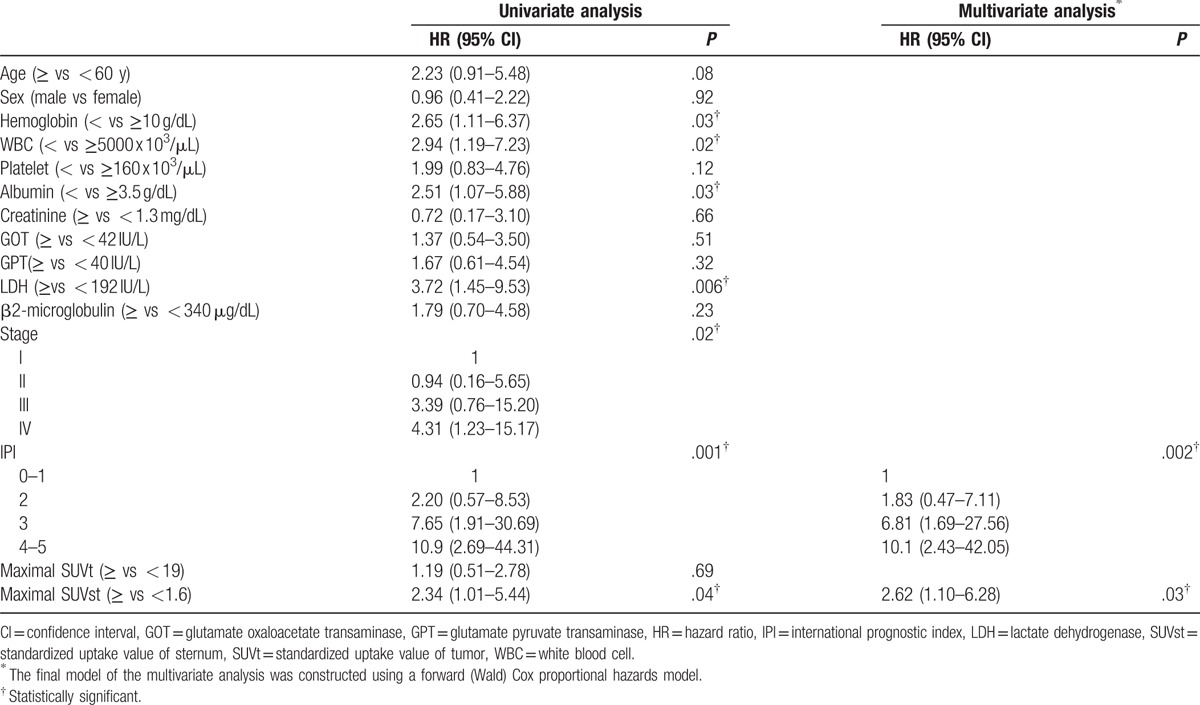
Cox proportional hazards model analysis of potential prognostic factors affecting overall survival.

## 4 Discussion

PET/CT using the tracer FDG incorporates metabolic tumor function with anatomic localization. In this study, the metabolic activity of the primary tumor was again confirmed to be a predictor for PFS. It, along with advanced stage and high LDH level, was associated with shorter PFS on univariate analysis; however, it was the only independent factor on multivariate analysis. It is not surprising that tumor SUV might correlate with patient survival, because previous studies have reported tumor SUV had a correlation with the Ki-67 proliferation index,^[17,18]^ a known prognostic factor in DLBCL.^[19]^ Chihara et al^[20]^ reported high SUV >30 was independently associated with shorter PFS. The result was similar to another study conducted by Miyazaki et al,^[21]^ but different SUV cutoff value of 15 was reported. In our result, PFS curves were not statistically significant with other SUV cutoff values except 19. To determine the appropriate SUV, cutoff value may need further multicenter analysis due to different PET/CT protocol, individual patient characteristics, and case numbers possibly affecting the result.

The increase of bone marrow metabolism depicted upon FDG PET/CT as a survival prognostic factor for malignant diseases has not been frequently discussed in the literature. Prévost et al^[14]^ studied a series of 120 patients with nonsmall cell lung cancer and found that bone marrow hypermetabolism along with FDG PET nodal stage and some hematologic parameters were significant independent predictors of mortality. Cicone et al^[15]^ also reported FDG uptake by bone marrow was significantly related to disease-free state and OS in patients suffering from squamous cell carcinoma of the head and neck. Haznedar et al^[22]^ concluded that PET/CT allows identification of high-risk myeloma patients; however, the extramedullary lesions with the highest SUV rather than bone marrow uptake independently predicted inferior OS. In the above 3 studies, the bone marrow SUV was all defined as the mean value of the 3 vertebrae (lumbar 3, 4, and 5 by Cicone et al^[15]^ and Haznedar et al,^[22]^ while Prévost et al^[14]^ did not mention the selected portion). In the literature review, however, the prognostic significance of sternal FDG uptake was rarely evaluated. The current study revealed that bone marrow hypermetabolism over the sternum was an independent predictive factor for OS in patients with DLBCL. Different biological characteristics of different tumor types may relate to the difference observed between our findings and the other studies.

Bone marrow hypermetabolism in malignancy is possibly associated with factors of both the host and the tumor itself. Secretion of stimulating cytokines such as interleukin-6, colony-stimulating factors, and vascular endothelial growth factor by the primary tumor may associate with activation of the hematopoietic process of bone marrow. It has been recognized that diffuse FDG uptake in the bone marrow, that is, above the image background, may be related to reactive myelopoiesis both in Hodgkin and NHL.^[23,24]^ Bone marrow micrometastases may also be another possible explanation of increased bone marrow metabolism. Well differentiation between reactive and pathologic diffuse infiltration due to lymphoma revealed with PET positivity may improve with experience.^[25]^ For host factors, coexisting illness such as relative hypoxemic states presented by decreased PaO_2_ and the hemoglobin level can some degree stimulate hematopoiesis and cause bone marrow hypermetabolism.

The prognostic impact of focal FDG abnormality in the bone marrow has remained unclear and controversial until now. Previously reported studies so far have had insufficient numbers to validate the outcome of this group separately.^[25–27]^ Cerci et al^[28]^ reported that, in patients with DLBCL, focal FDG-avid bone marrow depicted on PET without positive histology have similar survival from that in patients without tumor spread to the marrow; meanwhile, patients with focal FDG abnormality on PET and positive bone marrow histology had significantly poorer survival. The current study confirmed that the sternal FDG uptake, which was not related to bone marrow involvement, clinical stage, and hematological parameters, played a role in predicting poor OS. However, further studies are required to better identify the biological or molecular mechanism of increased bone marrow FDG uptake, and its relevance to patient outcome in DLBCL.

There are some limitations in the present study. The most important ones are inherent to a retrospective design as well as the small patient population. Another limitation of studies regarding DLBCL is the inclusion of different histological subtypes. Further confirmatory results may be conducted with a prospective study design, larger study population, and a more specific histological subtype collection.

## 5 Conclusion

Pending external validation, our result suggested that elevated maximal SUVst ≥1.6 was a predictor of shorter OS, independent of IPI score. Besides, an elevated maximal SUVt ≥19 was shown once again to be a predictor of shorter PFS, similar to some previous studies.

## Supplementary Material

Supplemental Digital Content
