# Supplementary material for: Tumor and bone marrow uptakes on [18F]fluorodeoxyglucose positron emission tomography/computed tomography predict prognosis in patients with diffuse large B-cell lymphoma receiving rituximab-containing chemotherapy
Source: Medicine (Baltimore). 2017 Nov 10;96(45):e8655. doi: 10.1097/MD.0000000000008655 (PMC5690797; doi:10.1097/MD.0000000000008655)

**Supplemental Content**

**Supplemental Figure Legends**

**Figure that illustrates the comparison of maximal SUVst between patients at different clinical stages by Kruskal-Wallis test.** Ninety-five percent confidence intervals for median of maximal SUVst at different stages were shown. Patients at stage IV seemed to have higher maximal SUVst; howerer, the difference didn’t meet statistically significant (*P*░=░.27).


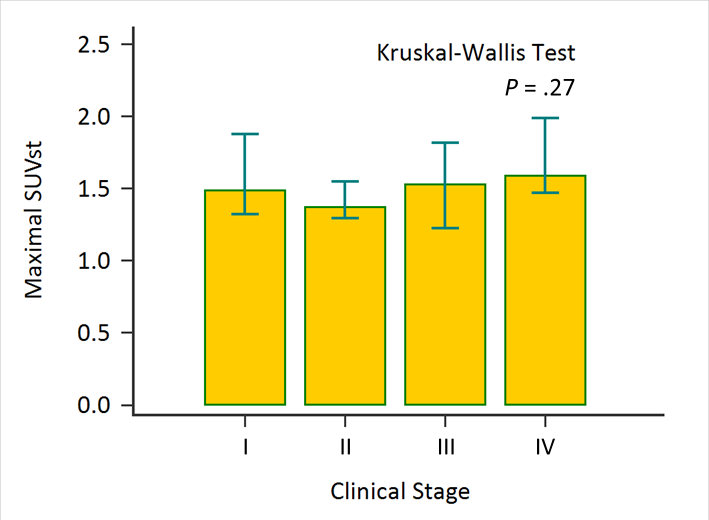

Supplement: Supplemental Digital Content [file medi-96-e8655-s001.doc]
